# Promises and limitations of deep learning for predicting knee osteoarthritis progression from medical imaging: A systematic review

**DOI:** 10.1002/ksa.70555

**Published:** 2026-08-03

**Authors:** Amna Gillani, Rüdiger von Eisenhart‐Rothe, Klaus Woertler, Michael T. Hirschmann, Daniel Rueckert, Florian Hinterwimmer

**Affiliations:** ^1^ Department of Orthopaedics and Sports Orthopaedics, School of Medicine and Health TUM University Hospital, Technical University of Munich Munich Germany; ^2^ Chair for AI in Healthcare and Medicine TUM University Hospital, Technical University of Munich Munich Germany; ^3^ Musculoskeletal Radiology Section TUM University Hospital, Technical University of Munich Munich Germany; ^4^ University Department of Orthopedic Surgery and Traumatology Kantonsspital Baselland Bruderholz Switzerland; ^5^ Department of Clinical Research, Research Group Michael T. Hirschmann, Regenerative Medicine & Biomechanics University of Basel Basel Switzerland; ^6^ Department of Computing Imperial College London London UK

**Keywords:** deep learning, disease progression, knee osteoarthritis, medical imaging, prediction models

## Abstract

**Purpose:**

To systematically evaluate the performance, methodological quality, and translational barriers of deep learning (DL) models for predicting knee osteoarthritis (KOA) progression from medical imaging.

**Methods:**

Following PRISMA guidelines, we searched PubMed, Scopus, and Web of Science (inception to June 2026) for peer‐reviewed studies applying DL to predict KOA progression from medical imaging. Two reviewers independently screened studies, extracted data, and assessed risk of bias using PROBAST‐AI. The primary outcome was area under the receiver operating characteristic curve (AUC). Narrative synthesis was performed due to heterogeneity in imaging modalities, outcome definitions, and prediction horizons.

**Results:**

From 1116 records, 33 studies (2019–2026) met the inclusion criteria, encompassing 50 predictive models. Sample sizes ranged from 340 to 52,981 knees (median: 4298). Nine progression definitions were identified and categorised as structural deterioration (*n* = 14), symptomatic worsening (*n* = 2), surgical endpoints (*n* = 8) or combined outcomes (*n* = 9). Nearly all studies (97%) used the Osteoarthritis Initiative dataset for training, and only 27% performed external validation. Surgery prediction models showed the highest descriptive median AUC (0.87), compared with structural (0.78) and symptomatic (0.79) progression models, but reflect non‐disease factors including access to care. MRI‐based models showed higher internal median AUCs (0.85) than radiograph‐based models (0.81), but none underwent external validation. Externally validated models showed performance degradation (median AUC: 0.75 vs. 0.81 internal). Combining imaging modalities did not consistently improve predictions. However, studies leveraging longitudinal trajectories reported higher internal performance. Most studies showed low risk of bias in participant selection and outcome assessment, but 76% had unclear or high risk in the analysis domain.

**Conclusions:**

DL models demonstrate proof‐of‐concept for predicting KOA progression but require substantial improvements before clinical deployment. Overcoming translational barriers requires standardised progression definitions integrating structural and symptomatic outcomes, rigorous multi‐site validation, and models that effectively leverage multimodal and longitudinal data.

**Level of Evidence:**

N/A.

**Abbreviations**
AUC: area under the receiver operating characteristic curve
DL: deep learning
IQR: interquartile range
KL: Kellgren–Lawrence grading system
KOA: knee osteoarthritis
KOOS: Knee Injury and Osteoarthritis Outcome Score
MOST: The Multicenter Osteoarthritis Study
OAI: The Osteoarthritis Initiative
PROBAST‐AI: Prediction model Risk Of Bias Assessment Tool for Artificial Intelligence
WOMAC: Western Ontario and McMaster Universities Arthritis Index

## INTRODUCTION

Knee osteoarthritis (KOA) is a progressive degenerative joint disease with increasing prevalence and economic burden [49]. Furthermore, it is one of the most common causes of pain, loss of function, and disability in adults [4]. Currently, KOA is often diagnosed late in the disease process, limiting the effectiveness of interventions such as weight loss, range of motion exercises, and physical strengthening, which are most effective in the early stages of the disease [5, 50]. Early detection of high‐risk individuals would also improve subject selection for clinical trials of disease‐modifying drugs [25].

Numerous predictive models for KOA progression have been developed to identify at‐risk individuals [48]. However, many studies rely primarily on basic demographic variables, such as age, gender, and body mass index (BMI). These models do not capture the heterogeneous nature of KOA progression [55] and exhibit poor performance in predicting disease onset in asymptomatic patients with underlying joint degeneration [31]. Prognostic models must incorporate imaging data to capture the structural and pathological changes that underlie disease progression.

The clinical evaluation of KOA traditionally relies on weight‐bearing radiographs to assess key indicators, such as osteophytes, subchondral sclerosis, and joint space narrowing [24]. These features form the basis of the Kellgren–Lawrence (KL) system, which is the gold standard for grading KOA severity [26]. However, KOA also involves changes in soft tissue, including cartilage, menisci, ligaments, synovial tissue, and fat [32], which cannot be visualised on radiographs. Therefore, magnetic resonance imaging (MRI) is also a valuable tool for studying the early stages of disease progression. Combining both modalities could provide a more comprehensive picture of the disease.

Large public datasets such as the Osteoarthritis Initiative (OAI) [36] and Multicenter Osteoarthritis Study (MOST) [53], with their multimodal imaging, comprehensive clinical assessments, and multi‐year follow‐ups, have facilitated numerous DL studies on KOA progression. Previous reviews have examined osteoarthritis severity classification and diagnosis [37, 51, 56, 62, 63, 66], non‐imaging data [48], specific imaging modalities [8, 35], or multiple joints [2, 6, 9]. However, none focused specifically on DL models using medical imaging to predict KOA progression.

We aim to evaluate the performance, methodological quality, and translational barriers of DL models for predicting KOA progression from medical imaging. We systematically reviewed the literature to answer the following questions: How is KOA progression defined? What imaging modalities and DL architectures are being employed, and how well do these models perform? Which clinical and technical barriers prevent clinical deployment?

## METHODS

The study was designed to conform to the Preferred Reporting Items for Systematic Reviews and Meta‐Analyses (PRISMA) guidelines [42]. The review protocol was registered prospectively in PROSPERO (registration number: CRD420251080953)

The following criteria determined study eligibility:

Inclusion criteria:

- Human studies involving KOA
- Use of at least one medical imaging modality
- Application of DL methods
- Prediction of future outcomes (disease progression)
- Written in the English language
- Peer‐reviewed journal or conference papers

Exclusion criteria:

- Studies including anatomical regions other than the knee
- Studies focusing only on the correlation and association of features with progression without predictive modelling
- Use of only pre‐computed radiomics or handcrafted features
- Cross‐sectional studies predicting current OA severity only
- Studies using omics data as primary predictors
- Conference abstracts, review articles, editorials and unpublished manuscripts

There was no restriction on studies based on geographic location, clinical setting, or patient demographics.

We searched PubMed, Web of Science, and Scopus databases from inception to 10 June 2026, and manually screened the reference lists of included articles for additional relevant studies. Search strategies combined controlled vocabulary and free‐text terms related to knee osteoarthritis, deep learning, disease progression, and medical imaging. Table 1 provides the complete search strategies for each database.

**Table 1:** Databases and search strings.

Database	Search string
PubMed	(“Osteoarthritis, Knee”[Mesh] OR “knee osteoarthritis”) AND (“Deep Learning”[Mesh] OR “Artificial Intelligence”[Mesh] OR “deep learning” OR “neural network*” OR “convolutional neural network*” OR “CNN”) AND (“Disease Progression”[Mesh] OR “Prognosis”[Mesh] OR prognosis OR progress* OR worsen* OR “future outcome*” OR “prediction”) AND (“Imaging”[Mesh] OR “Radiography”[Mesh] OR “Magnetic Resonance Imaging”[Mesh] OR x‐ray* OR radiograph* OR MRI)
Scopus	TITLE‐ABS‐KEY((“knee osteoarthritis” OR “knee OA”) AND (“deep learning” OR “artificial intelligence” OR “neural networks” OR “convolutional neural network*” OR CNN) AND(“disease progression” OR prognosis OR progress* OR worsen* OR “future outcome*” OR predict*) AND (“x‐ray” OR “radiograph*” OR “MRI” OR “medical imaging” OR “radiology”))
Web of Science	TS = ((“knee osteoarthritis” OR “knee OA”) AND (“deep learning” OR “artificial intelligence” OR “neural networks” OR “CNN” OR “convolutional neural network*”) AND (“disease progression”OR prognosis OR progress* OR worsen* OR “future outcome*“ OR predict*) AND (“x‐ray” OR “radiograph*” OR “MRI” OR “medical imaging” OR “radiology”))

Two reviewers (A.G., F.H.) independently screened titles and abstracts according to the predefined criteria. The same reviewers subsequently performed full‐text screening of all potentially eligible studies. At each stage, reviewers resolved disagreements through discussion, consulting a third reviewer when necessary.

Data were independently extracted by the reviewers using a standardised form. Missing information was recorded as “not available” (N/A), and study authors were not contacted for additional data. For each included study, the following information was systematically collected:

- Publication details: First author, publication year, title, journal
- Study population: Dataset name, number of subjects/images (total and per class), inclusion/exclusion criteria
- Outcome definition: Type of progression (structural, symptomatic, surgical), specific definition, and criteria used
- Imaging details: Modality, acquisition parameters, preprocessing steps
- Additional data: Other modalities or features used (clinical, demographic data)
- Model characteristics: DL architecture, input format, external pretraining details
- Training methodology: Data splitting strategy, augmentation techniques, sampling strategies, loss functions, optimiser settings, learning rate, and epochs
- Prediction details: Target variable, prediction horizon
- Performance metrics: Area under curve (AUC), accuracy, sensitivity, specificity, precision, recall, balanced accuracy
- Methodological quality and Reproducibility: Use of interpretability methods, baseline comparisons, clinical comparisons, statistical significance testing, code availability

We assessed the risk of bias using the Prediction model Risk Of Bias Assessment Tool for Artificial Intelligence (PROBAST‐AI 2025 version) [38], which evaluates bias and applicability across four key domains: participants, predictors, outcome, and analysis. Each domain was rated as low, high, or unclear risk of bias based on the criteria defined in PROBAST‐AI. Studies were considered at an overall high risk of bias if any domain was rated as high risk. Both reviewers independently conducted risk of bias assessments, resolving disagreements through discussion and, when necessary, consultation with a third reviewer.

To assess whether model performance varied systematically across study characteristics, we conducted subgroup analyses of all models that reported AUC, the primary effect measure. The unit of analysis was the individual predictive model. Where a study reported multiple models for the same outcome and time horizon, only the best‐performing model by AUC was extracted. Where a study predicted outcomes at multiple time horizons, the best‐performing model at each time horizon was extracted separately, and the prediction time horizon was recorded in years. Where a study reported both internal and external validation, both results were extracted and flagged accordingly.

A formal quantitative meta‐analysis was not performed due to the heterogeneity in imaging modalities, outcome definitions, DL methods, and prediction horizons. Studies frequently reported multiple correlated model‐level AUCs without confidence intervals or standard errors, and many results were derived from overlapping patient samples due to the widespread use of the OAI dataset. Consequently, a narrative synthesis was planned, grouping studies by progression type, imaging modality, and prediction timeframe.

## RESULTS

The systematic search identified a total of 1116 records across the three databases. After removing duplicates and screening titles, abstracts, and full texts, 33 studies met the inclusion criteria [1, 3, 7, 10, 11, 12, 13, 15, 16, 17, 18, 20, 21, 22, 23, 28, 29, 30, 34, 40, 41, 43, 44, 45, 46, 47, 52, 57, 58, 59, 60, 64, 65]. Figure 1 shows the flow diagram for the study selection process.

**Figure 1:**
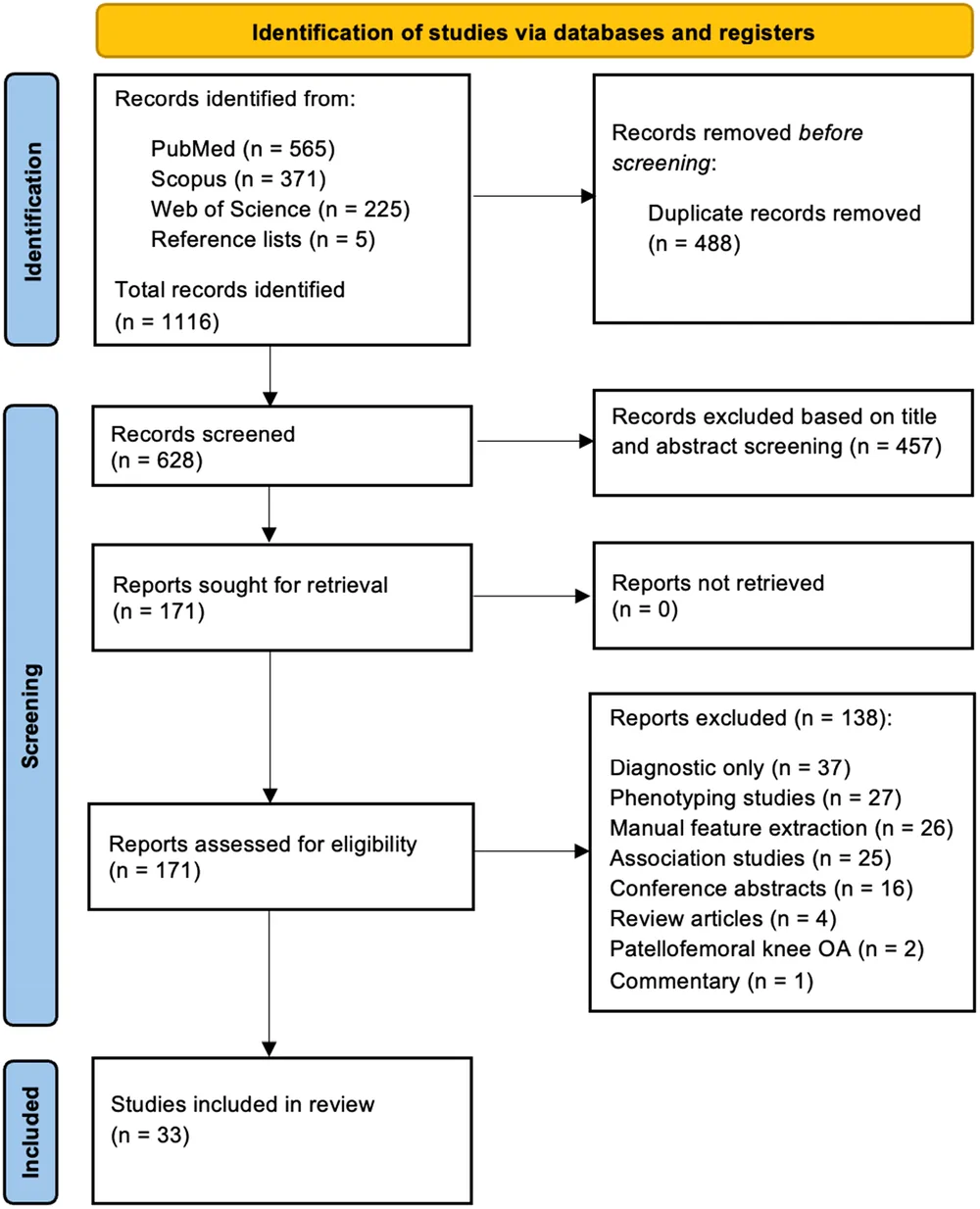
PRISMA flow diagram for study selection process.

### Study characteristics and outcome measures

The included studies were published between 2019 and 2026. Sample sizes ranged from 340 to 52,981 knees (median: 4298, interquartile range [IQR]: 1246–8953). Studies employed nine distinct definitions for disease progression. We grouped the studies into the following categories based on their progression definitions:

- Structural progression (*n* = 14 studies)
  Structural changes were the most studied outcome. Most studies defined KOA progression as a KL grade increase (*n* = 8), followed by joint space narrowing (JSN; *n* = 4), and the first appearance of a KL grade ≥ 2 (*n* = 2).
- Symptomatic progression (n = 2 studies)
  Symptomatic progression was the least studied outcome and depended on changes in patient reported outcome measures (PROMs). One study used a ≥ 9‐point increase in the Western Ontario and McMaster Universities Arthritis Index (WOMAC) score over 48 months, while the other study considered worsening Knee Injury and Osteoarthritis Outcome Score (KOOS) pain over 8 years.
- Surgical endpoint (*n* = 8 studies)
  Total knee replacement (TKR) was the primary surgical endpoint, with prediction horizons ranging from 12 months to 9 years. Two studies modelled time‐to‐TKR using survival analyses, while one study included uni‐compartmental knee replacement.
- Combined outcomes (*n* = 9 studies)
  Five studies combined structural and symptomatic criteria (an increase in KL grade or JSN accompanied by an increase in WOMAC pain scores), and four studies combined structural and surgical definitions (an increase in KL grade or TKR).

Table 2 provides detailed progression definitions and outcome time horizons.

**Table 2:** Study and data set characteristics.

Progression type	Study	Year	data set	Sample size	Progress definition	Outcome time
Structural	Almhdie‐Imjabbar et al. [3]	2022	OAI and MOST	OAI: 2740 images, 425 subjects; MOST: 845 images, 297 subjects	Increase in OARSI medial JSN grades	OAI: 48 months; MOST: 60 months
	Butler et al. [7]	2025	OAI	47,927 images, 4796 subjects	Increase in KL grade	1 year
	Chen et al. [11]	2025	OAI	2164 images, 700 knees	Appearance of KL grade >=2	4 years
	Guan et al. [15]	2020	OAI	1950 images	>=0.7 mm decrease in medial JSW	2 years
	Guo et al. [17]	2025	OAI	11,682 images, 1947 subjects	Reduction in fixed JSW measured at multiple points	6 years
	Han et al. [18]	2022	OAI and MOST	OAI: 52,981 images, 4796 subjects; MOST: 19,340 images, 3015 subjects	Increase in KL grade	Up to 8 years
	Hu et al. [23]	2022	OAI	3,294 images	Longitudinal KL grades	Up to 2 years
	Lee et al. [29]	2023	OAI	23,958 images, 2328 subjects	KL grade >=2	8 years
	Panfilov et al. [43]	2022	OAI	4866 knees, 2702 subjects	Increase in KL grade	8 years
	Panfilov et al. [44]	2025	OAI	2421 to 3967 subjects depending on timepoint	Increase in KL grade	1, 2, 3, 4 and 8 years
	Schiratti et al. [52]	2021	OAI	9280 images, 3268 subjects	At least 0.5 mm decrease in JSW	12 months
	Theocharis et al. [57]	2025	OAI	6228 images, 3114 subjects	Increase in KL grade	1, 2, 3 and 4 years
	Wang et al. [60]	2025	OAI, Private Data	OAI: 5565 images; Private Data: 274 images	Increase in KL Grade	4 years
Symptomatic	Guan et al. [16]	2022	OAI	5000 images	9 point or greater increase in WOMAC score	2 years
	Lee et al. [28]	2021	OAI	3838 images, 2799 subjects	Worsening KOOS pain	8 years
Surgical outcome	Cigdem et al. [12]	2024	OAI, MOST, Private Data	OAI: 1681 images; MOST: 682 images	TKR	Up to 9 years (OAI)3 years (Internal data set)
	Cigdem et al. [13]	2025	OAI, MOST, Private Data	OAI: 850 knees, 547 subjects; MOST: 754 knees, 518 subjects; Internal Dataset: 164 knees	TKR	Up to 9 years
	Houserman et al. [20]	2023	Private Dataset	8301 images, 2767 subjects	TKR or UKA	N/A
	Leung et al. [30]	2020	OAI	364 case control pairs	TKR	Within 9 years
	Rajamohan et al. [46]	2023	OAI and MOST	353 case control pairs	TKR	Within 9 years (OAI); Within 7 years (MOST)
	Rajamohan et al. [45]	2025	OAI and MOST	OAI: 364 case control pairs; MOST: 574 case control pairs	TKR	At 1, 2 and 4 year time points
	Tolpadi et al. [59]	2020	OAI	35,382 images, 4796 subjects	TKR	Within 5 years
	Zhang et al. [65]	2025	OAI and MOST	OAI: 353 case control pairs, MOST: 270 case control pairs	TKR	Up to 9 years (OAI); Up to 7 years (MOST)
Combined	Ahadian et al. [1]	2025	OAI	600 subjects, 340 images	Loss in mJSW greater than 0.7 mm and increase in 9 points in WOMAC score	2–4 years
	Chen et al. [10]	2024	OAI	4796 subjects	WOMAC pain increase of 5 points and increase in KL grade	2 years
	Hu et al. [22]	2023	OAI	182 case control pairs	Loss in medial mJSW greater than 0.7 mm and increase in 9 points in WOMAC score	2–4 years
	Hu et al. [21]	2025	OAI	600 subjects	Loss in medial mJSW greater than 0.7 mm and increase in 9 points in WOMAC score	2–4 years
	Lv et al. [34]	2025	OAI	600 subjects	Loss in medial mJSW greater than 0.7 mm and increase in 9 points in WOMAC score	4 years
	Nguyen et al. [41]	2022	OAI	4796 subjects	Increase in KL grade or TKR	Up to 8 years
	Nguyen et al. [40]	2024	OAI	8953 images, 4796 subjects	Increase in KL grade, increase in OARSI grades or TKR	Up to 8 years
	Rajamohan et al. [47]	2026	OAI and MOST	OAI: 2248 images, MOST: 1079 images	Appearance of KL grade >=2; increase in KL grade or TKR	4 years (OAI); 5 years (MOST)
	Tiulpin et al. [58]	2019	OAI and MOST	OAI: 4928 knees, 2711 subjects, MOST: 3918 knees, 2129 subjects	Increase in KL grade or progression to TKR	Up to 8 years (OAI) 7 years MOST)
	Yin et al. [64]	2024	OAI	12,650 images, 3583 subjects	Increase in KL grade or TKR	Up to 8 years

Abbreviations: JSN, joint space narrowing; JSW, joint space width; KL, Kellgren–Lawrence Grade; KOOS, Knee injury and Osteoarthritis Outcome Score; mJSW, minimum joint space width; MOST, Multicenter Osteoarthritis Study; OAI, Osteoarthritis Initiative; OARSI, Osteoarthritis Research Society International; TKR, total knee replacement; UKA, uni‐compartmental knee arthroplasty; WOMAC, Western Ontario and McMaster Universities Arthritis Index.

## DATA AND MODELS

Beyond outcome heterogeneity, studies varied considerably in their imaging modalities and modelling approaches. Nearly all studies (*n* = 32, 97%) used the OAI dataset for training, while one study used a private dataset for training. Two studies trained models on both OAI and MOST.

Studies most frequently used radiographs (*n* = 16 studies, 48%), followed by MRI (*n* = 9, 27%), and multimodal approaches combining both MRI and radiographs (*n* = 8, 24%). All radiographic studies except one used the posteroanterior fixed flexion view, with a single study using three‐view radiographs. MRI studies commonly employed multiple acquisition sequences, with three‐dimensional double‐echo steady‐state (3D‐DESS; *n* = 12) and turbo spin‐echo (TSE; *n* = 13) sequences being the most prevalent. Twenty‐three studies (70%) incorporated clinical variables with imaging. The most frequently used clinical variables were age, sex, BMI, and baseline pain scores.

Convolutional neural networks (CNNs) were the predominant architecture type (*n* = 20 studies, 61%). Eight studies (24%) used Transformers, and three studies (9%) employed Graph Neural Networks (GNNs). One study used a variational autoencoder, and one study used a large language model (LLM) agent. Twenty‐two (67%) studies used only baseline data for prediction while eleven used data from multiple time‐points. Of these, eight treated each time point as an independent observation, while only three analysed the data longitudinally. Four studies predicted outcomes at multiple future time points, three conducted time‐to‐event analyses, one generated future images, three used regression, and the remainder focused on classification at a single future time point.

AUC was the most reported metric (*n* = 28 studies). Other commonly reported metrics include sensitivity, specificity, and balanced accuracy. Table 3 presents performance metrics and detailed technical implementations.

**Table 3:** Imaging, model and results summary.

Study	Imaging modality	Other modality	Model type	Model architecture	AUC	Other metrics	External validation performance	Interpretability
Almhdie‐Imjabbar et al. [3]	Radiographs	Clinical variables	Classification	Siamese CNNs + Trabecular bone texture analysis	OAI: 0.75; MOST: 0.81	Acc: > 0.80 (OAI); Acc> 0.70 (MOST)	AUC: 0.70‐0.74	Feature importance using variogram‐based fractal analysis
Butler et al. [7]	Radiographs	N/A	Classification	Variational autoencoder, diffusion model	0.71	Sens: 0.83; Spec: 0.51; F1 Score: 0.55	N/A	Knee landmark localisation generated by diffusion model
Chen et al. [11]	MRI (Sagittal 3D DESS)	Clinical variables	Classification	Shallow neural network, LASSO logistic regression	0.85	Sens: 0.75; Spec: 0.77; Acc: 0.76	N/A	LASSO for feature importance
Guan et al. [15]	Radiographs	Demographic and radiographic risk factors	Classification	DenseNet + FCN	0.863	Sens: 0.805; Spec: 0.805	N/A	Saliency maps
Guo et al. [17]	Radiographs	Clinical data	Regression	TransUNet (segmentation) and XGBoost (prediction)	N/A	*R* ^2^ = 0.804; MAE = 0.48; RMSE = 0.697; ICC = 0.887	N/A	SHAP for feature importance
Han et al. [18]	Radiographs	N/A	Classification	StyleGAN + nearest neighbour; ResNet‐50	0.69	Sens: 0.516; Spec: 0.886	AUC: 0.64	Saliency maps
Hu et al. [23]	Radiographs	N/A	Classification	Adversarial evolving neural network with VGG‐19, ResNet‐50 and ViT backbones	0.637	Overall acc: 0.627; MAE = 0.117	N/A	Evolution traces, t‐SNE
Lee et al. [29]	MRI (coronal and sagittal) radiographs	Clinical variables	Survival analysis	CNN + Cox proportional hazards model	N/A	Survival model C‐Index: 0.69	N/A	Survival curves for different phenotypes
Panfilov et al. [43]	MRI DESS	N/A	Classification	2D CNN encoder + Transformer	0.78	AP: 0.58, Multiclass BA: 0.52	0.78	N/A
Panfilov et al. [44]	Radiographs MRI 3D DESSMRI TSEMRI T2	Clinical variables	Classification	Multimodal transformers with CNN feature extractor	0.70–0.76	AP: 0.13–0.54	N/A	Relative utilisation rates using feature ablation
Schiratti et al. [52]	MRI COR IW TSE	Clinical variables	Classification	EfficientNet‐B0 feature extractor with attention + MLP	0.65	Precision: 0.13, Recall: 0.84	N/A	Attention maps
Theocharis et al. [57]	MRI 3D DESS	Clinical variables	Classification	Hypergraph CNN + graph predictor network	0.94 (Year 1), 0.89 (Year 2), 0.85 (Year 3), 0.80 (Year 4)	BA: 0.919 (y1), 0.881 (y2), 0.844 (y3), 0.794 (y4)	N/A	N/A
Wang et al. [60]	Radiographs	Clinical variables	Classification	Vision transformer encoder + MLP head	0.808	Sens: 0.918; Spec: 0.710; Acc: 0.741	AUC: 0.709Sens: 0.722Spec: 0.703	Attention maps
Guan et al. [16]	Radiographs	Clinical variables	Classification	CNN + ANN + FCN	0.807	Sens: 0.723, Spec: 0.809	N/A	Saliency maps
Lee et al. [28]	MRI SAG IW TSE	Clinical variables	Classification	3D DenseNet121	0.794	Sens: 0.498, Spec: 0.851, Acc: 0.810	N/A	N/A
Cigdem et al. [12]	Radiographs, MRI (DESS), MRI (TSE)	Clinical variables, image assessments	Survival Analysis	3D ResNet18, 2D ResNet18, RSF with Lasso Cox feature selection	0.945	Acc: 0.752; C‐Index: 0.853	MOST: Acc: 0.485Internal data: Acc: 0.555	Saliency maps
Cigdem et al. [13]	Radiographs, MRI (DESS), MRI (TSE)	Clinical variables, image assessments	Survival analysis	3D ResNet18, 2D ResNet18, RSF with Lasso Cox feature selection	0.820	Acc: 0.732; C‐Index: 0.773	MOST: Acc: 0.485Internal data: Acc: 0.555	Feature Importance
Houserman et al. [20]	3 View radiographs	N/A	Classification	EfficentNetB4	TKA: 0.97; UKA: 0.96	Acc: 0.878	N/A	N/A
Leung et al. [30]	Radiographs	N/A	Classification	ResNet34	0.87	Sens: 0.83; Spec: 0.77	N/A	Grad‐CAM
Rajamohan et al. [46]	MRI (coronal IW, sagittal IW, sagittal DESS) radiographs	Clinical variables	Classification	3D CNNs + 2D CNNs + MLP ensemble model	0.90	Sens: 0.80; Spec: 0.85	AUC: 0.65‐0.69	Saliency maps
Rajamohan et al. [45]	MRI (IW‐TSE) radiographs	N/A	Classification	ResNet + progressive risk formulation	0.87 (Year 1), 0.85 (Year 2), 0.86 (Year 4)	AUPRC:0.47 (Year 1), 0.63 (Year 2), 0.75 (Year 4)	AUC:0.77 (year 1), 0.79 (year 2), 0.81 (year 4)	N/A
Tolpadi et al. [59]	MRI (3D‐DESS) radiographs	Clinical variables	Classification	3D DenseNet‐121 + 2D DenseNet‐121 Ensemble	0.89	Acc: 0.785, Sens: 0.818, Spec: 0.784	N/A	Occlusion maps
Zhang et al. [65]	MRI (coronal IW‐TSE, sagittal IW‐TSE‐FS, coronal STIR, sagittal PD‐FS)	N/A	Classification	Vision transformer	OAI: 0.91; MOST: 0.82	Sens: 0.84; Spec: 0.80	N/A	Attention maps
Ahadian et al. [1]	Radiographs	Clinical variables	Classification	LLM Agent, MLP + CNN	0.82	Spec: 0.88; Acc: 0.82	N/A	Chain of thought reasoning
Chen et al. [10]	Radiographs MRI (DESS)	Clinical variables	Classification	Unified Mult‐modal multi‐view transformer	Pain: 0.714KL: 0.903	AP: 0.358 (pain), 0.73 (KL), Macro Acc: 0.654 (pain), 0.680 (KL)	N/A	Post‐hoc ablation analysis
Hu et al. [22]	MRI (4 sequences)	Clinical variables	Classification	3D DenseNet169	Baseline: 0.641 year: 0.7392 years: 0.775	Sens: 0.588, 0.632, 0.665; Spec: 0.660, 0.709, 0.715	N/A	Grad‐CAM
Hu et al. [21]	MRI 2D IW‐TSE‐FSMRI 3D‐DESS	Clinical variables, radiologist scores	Classification	Temporal‐regional graph CNN	0.856	N/A	N/A	GNNExplainer
Lv et al. [34]	MRI 2D IW‐TSE‐FSMRI 3D‐DESS	Clinical variables	Classification	Graph Convolution Network	0.85	Sens: 0.85, Spec: 0.69	N/A	GNNExplainer
Nguyen et al. [40]	Radiographs	Clinical variables	Ordinal Classification	Multi‐agent transformer network	N/A	BA: 0.55‐0.60	N/A	Attention maps
Nguyen et al. [41]	Radiographs	Clinical variables	Ordinal Classification	Resnet‐18 feature extractor + multi‐agent transformer framework	N/A	BA: 0.553, 0.537, 0.501, 0.475, 0.285, 0.271	N/A	Attention maps
Rajamohan et al. [47]	Radiographs	N/A	Classification	Resnet‐34 with attention	0.750	AUPRC: 0.456BA: 0.751	AUC: 0.764AUCPRC: 0.594BA: 0.675	N/A
Tiulpin et al. [58]	Radiographs	Clinical variables	Classification	CNN feature extractor + gradient boosting	0.81	AP: 0.70	AUC: 0.79	Attention maps
Yin et al. [64]	Radiographs	N/A	Classification	Dual input CNN with cross attention	Test 1: 0.761; Test 2: 0.746	Sens: 0.665, 0.675; Spec: 0.774, 0.738	N/A	Grad‐CAM

Abbreviations: Acc, accuracy; ANN, artificial neural network; AP, average precision; AUC, area under the receiver operating characteristic curve; BA, balanced accuracy; C‐Index, concordance index; CI, confidence interval; CNN, convolutional neural network; DESS, double‐echo steady state; FCN, fully convolutional network; FS, fat suppressed; GNN, graph neural network; Grad‐CAM, gradient‐weighted class activation mapping; ICC, intraclass correlation coefficient; IW, intermediate‐weighted; KL, Kellgren–Lawrence; LASSO, least absolute shrinkage and selection operator; MAE, mean absolute error; MLP, multilayer perceptron; MOST, Multicenter Osteoarthritis Study; OAI, Osteoarthritis Initiative; PD, proton density; *R* ^2^, coefficient of determination; RMSE, root mean square error; RSF, random survival forest; Sens, sensitivity; SHAP, Shapley additive explanations; Spec, specificity; STIR, short tau inversion recovery; t‐SNE, t‐distributed stochastic neighbour embedding; TKA, total knee arthroplasty; TSE, turbo spin echo; UKA, uni‐compartmental knee arthroplasty; ViT, vision transformer.

### Subgroup analysis

To assess whether model performance varied systematically across different study characteristics, we conducted subgroup analyses to assess differences in model performance across image modalities, clinical outcomes, prediction horizons, and validation strategies. Since multiple progression definitions were used within each category, subgroup analyses should be considered descriptive and exploratory; within‐group heterogeneity should be taken into account when interpreting differences between subgroups.

We extracted 50 predictive models from the 28 studies that reported AUC. Median and interquartile ranges (IQR) are reported for each subgroup. We did not perform formal statistical comparisons as many models were developed using the same underlying dataset, and multiple models were often reported within the same studies, which violates independence assumptions.

### By imaging modality

MRI‐based models (*n* = 13 from 9 studies) demonstrated the highest internal AUC, with a median AUC of 0.85 followed by multimodal models combining MRI and radiographs (*n* = 14 from 7 studies) with a median AUC of 0.84 while radiograph‐only models (*n* = 13 from 12 studies) showed the lowest internal AUC, with a median AUC of 0.81. Notably, none of the MRI models were externally validated. For other modalities, models consistently reported lower AUCs on external validation compared to internal validation. Figure 2 shows the AUCs of the models stratified by imaging modality.

**Figure 2:**
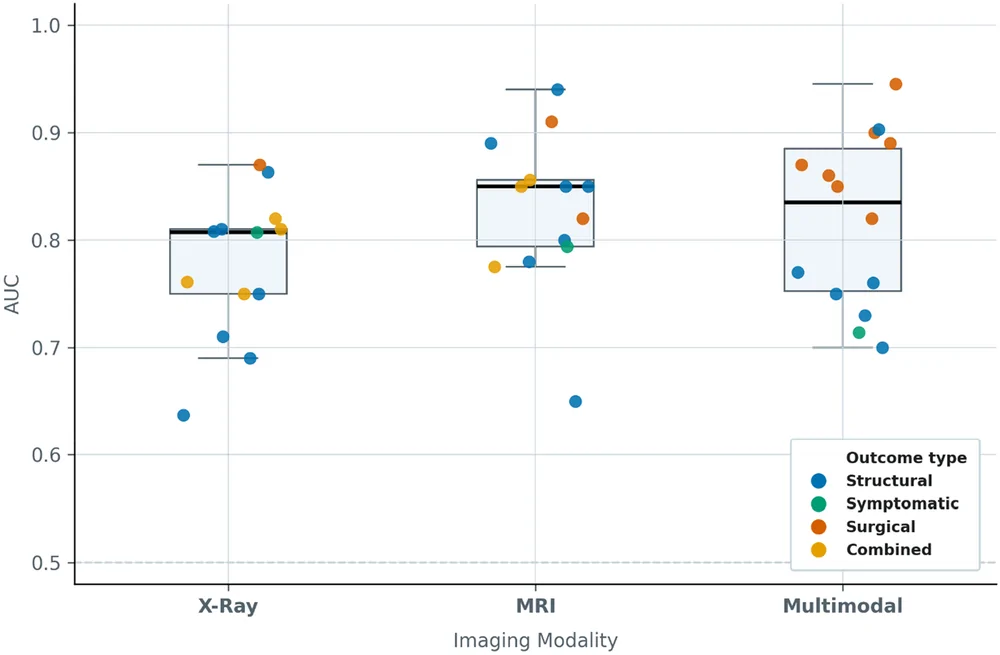
Distribution of AUC values across imaging modalities. Strip plot showing the internal AUC of all deep learning models stratified by imaging modality (X‐ray, MRI, and multimodal). Each point represents one predictive model, colour‐coded by progression type (structural, symptomatic, surgical, or combined). The box and whisker plot denotes the median, interquartile range, and full range of AUC values per subgroup. Differences in median AUC across modalities should be interpreted descriptively. AUC, area under the receiver operating characteristic curve; MRI, magnetic resonance imaging.

### By progression type

Models predicting surgical outcomes (*n* = 10 from 7 studies) had the highest median AUC of 0.87, followed by models predicting combined outcomes (*n* = 7 from 7 studies; median AUC 0.81). Models predicting structural progression (*n* = 20 from 12 studies; median 0.78) showed similar performance to models predicting symptomatic deterioration (*n* = 3 from 3 studies, median 0.79). Within each outcome group, externally validated models again showed lower AUCs compared with internally validated ones. Notably, none of the models measuring combined outcomes were externally validated. Figure 3 shows the AUCs of the models stratified by clinical outcomes.

**Figure 3:**
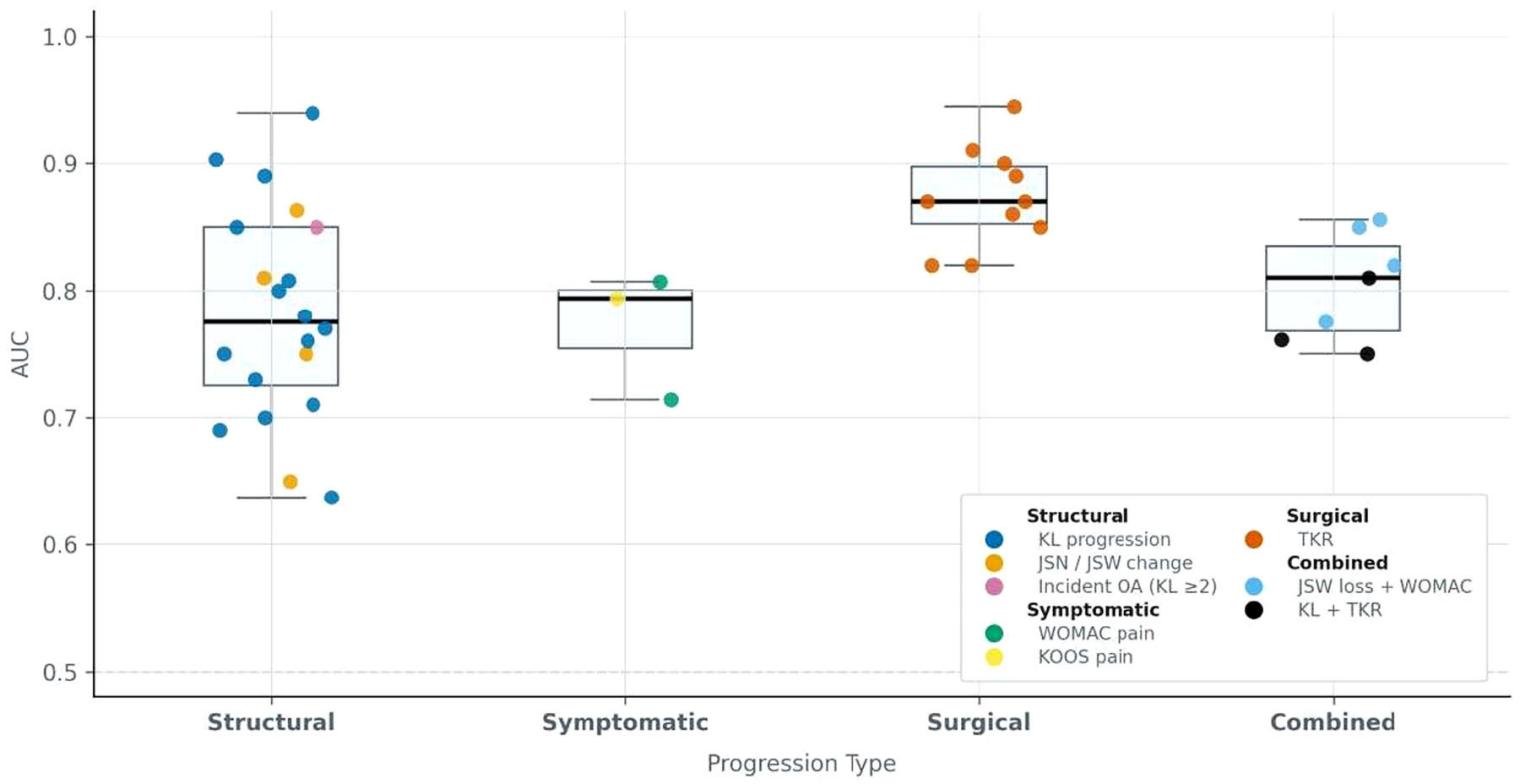
Distribution of AUC values across progression types. Strip plot showing the internal AUC of all deep learning models stratified by progression category (structural, symptomatic, surgical, and combined). Each point represents one predictive model, colour‐coded by specific progression definition. The box and whisker plot denotes the median, interquartile range, and full range of AUC values per subgroup. Differences in median AUC across progression types should be interpreted descriptively. AUC, area under the receiver operating characteristic curve.

### By outcome time

Models predicting short‐term outcomes (≤2 years; *n* = 10 from 6 studies), intermediate outcomes (2–5 years; *n* = 18 from 15 studies), and long‐term outcomes (>5 years; *n* = 12 from 11 studies) showed comparable median AUCs of 0.81 (IQR: 0.72–0.89), 0.81 (IQR: 0.76–0.85), and 0.82 (IQR: 0.78–0.88), respectively.

## METHODOLOGICAL QUALITY

Nine studies (27%) performed external validation. Eight validated models on the MOST dataset, two of which additionally validated on a private dataset, while one study validated exclusively on a private dataset. Five (15%) studies performed validation on a hold‐out test centre of OAI, which cannot be considered truly independent as these sites share standardised protocols and quality control. The remaining 19 (58%) studies did not conduct any external validation. Overall, across all models, those validated internally (*n* = 40) had a median AUC of 0.81 (IQR: 0.76–0.86; range: 0.64–0.95), whereas externally validated models (*n* = 10) had a lower median AUC of 0.75 (IQR: 0.71–0.79; range: 0.64–0.81). This performance degradation was most pronounced for surgical outcome prediction (internal median: 0.87 vs external: 0.78).

Most studies (*n* = 28, 85%) included some form of interpretability such as attention and saliency maps, GNN Explainer, Least Absolute Shrinkage and Selection Operator (LASSO), and SHapley Additive exPlanations (SHAP) for feature importance. Only 15 studies (45%) provided publicly available source code, and three studies provided links to empty repositories.

We assessed risk of bias using PROBAST‐AI [38] for model development and evaluation across four domains: participants, predictors, outcome, and analysis. Most studies demonstrated low risk of bias in participant selection due to the use of well‐established datasets (OAI, MOST). Three studies showed a high risk of bias because they restricted inclusion to patients who received TKR, creating unrepresentative datasets. The predictor and outcome domains demonstrated a low risk of bias across all studies due to standardised imaging protocols and patient cohorts, and clear outcome criteria. In the model evaluation category, the analysis domain presented the highest risk of bias (20 studies unclear, 5 studies high risk). The primary concerns were lack of external validation, inadequate sample sizes relative to the complexity of the deep learning model, and potential overfitting. Figure 4 illustrates the risk‐of‐bias plot, as evaluated using PROBAST‐AI.

**Figure 4:**
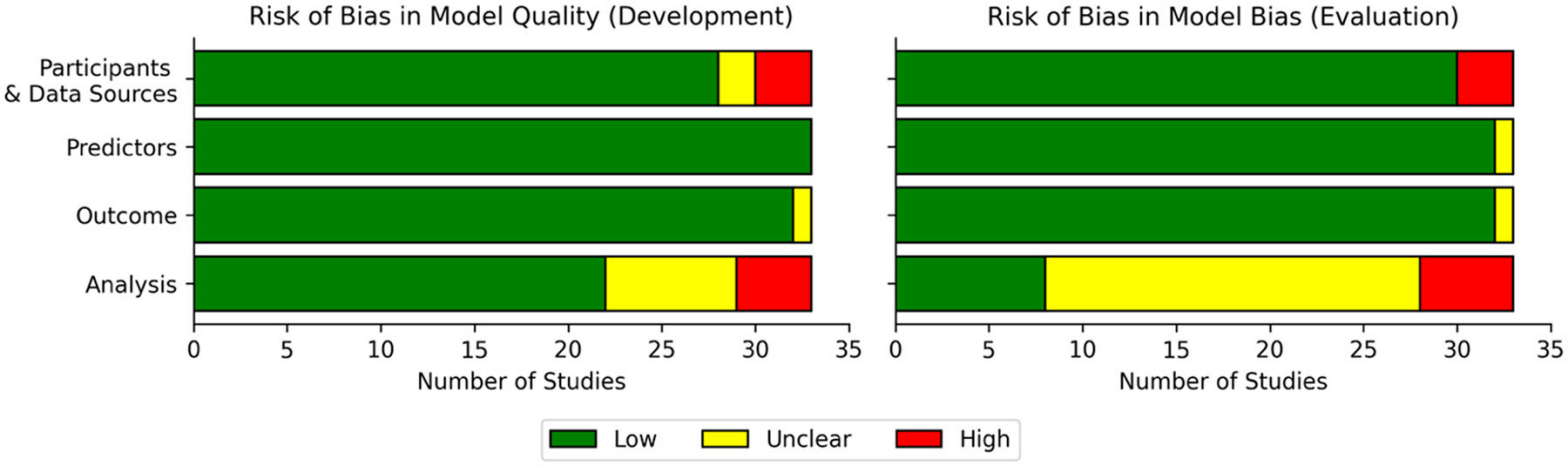
Risk of bias over four domains assessed using PROBAST‐AI. Left: risk of bias in the development phase; right: risk of bias in the evaluation phase.

## DISCUSSION

DL models for predicting KOA progression from medical imaging show promising proof‐of‐concept performance, but are undermined by heterogeneous outcome definitions, overwhelming reliance on a single dataset, and scarce external validation. Current prediction targets fail to capture both the structural and symptomatic aspects of the disease. Across 33 studies, we identified nine distinct definitions of KOA progression, focusing on structural changes, surgical interventions, and, less frequently, patient symptoms. Each approach captures only one facet of this multifaceted disease.

Most studies focused solely on structural worsening, despite evidence that radiographic KOA has a weak correlation with knee pain [19]. This is a significant gap because pain is the hallmark of KOA, which drives patients to seek medical attention and reduces their quality of life [39]. Although KL grades are the gold standard for assessing KOA severity, they lack sensitivity to fine‐grained joint changes and depend on radiologist interpretation. Only three studies [10, 16, 28] predicted pain progression, but they differed substantially in outcome definition and prediction horizons and did not account for any treatment undergone by patients. Four studies examined pain alongside structural change, but only when the two were correlated, and none were externally validated. Notably, no study examined cases where symptomatic worsening occurs without structural progression, or vice versa. In contrast to the limited attention to symptomatic outcomes, surgical endpoints were examined by seven studies, demonstrating the highest performance (median AUC: 0.87). However, surgery is not an appropriate indicator of disease progression as it depends on multiple non‐disease factors, including comorbidities, insurance status, access to care, and race [27].

MRI‐based methods showed higher internal median AUCs than radiograph‐based approaches in predicting progression (median AUC 0.85 vs. 0.81). This reflects the increased sensitivity of MRI in detecting early changes, such as cartilage defects, meniscal abnormalities, and bone marrow lesions, that precede changes visible on radiograph [14]. However, none of the MRI‐based models were externally validated, which raises concerns about generalisability. Given the higher cost and limited availability of MRI compared to radiography, these models must first show reliable external performance and offer a clear advantage in prediction to justify their use in clinical settings.

Combining MRI with radiographs should theoretically improve predictions, as it captures more information than either modality alone. However, multimodal models did not consistently outperform unimodal models. Panfilov et al. reported that MRI alone matched or exceeded multimodal approaches, while Hu et al. found that models trained on a single MRI sequence performed comparably to those using four MRI sequences [22, 44]. Similarly, several studies found that the addition of clinical data did not improve model performance [3, 22, 34].

Modality competition [61] explains some failures. Panfilov et al. demonstrated that their multi‐modal model ignored radiographic data [52]. In this case, the richer 3D MRI features likely overshadowed the sparser 2D radiograph features during optimisation. Although MRI and radiographs provide complementary information, some studies have used both on tasks such as KL grade progression, where only one modality is sufficient for prediction [29, 44]. In contrast, multimodal models showed higher median AUCs than unimodal approaches in predicting surgical outcomes [12, 13, 46], where decisions depend on multiple factors for which different modalities provide genuinely complementary information. Interestingly, no study utilised CT imaging, which has been shown to have higher sensitivity than conventional radiography in detecting structural features of KOA [54], but is absent from the OAI.

Only three studies analysed longitudinal data. The rest relied on baseline imaging alone or treated images from different time points as independent observations. This choice discards information about the disease trajectory. All three studies using longitudinal data demonstrated its value. Chen et al. demonstrated that incorporating temporal observations (0–72 months) consistently improved prediction performance for both WOMAC pain and KL grade [10]. Similarly, Guo et al. showed that using longitudinal JSW measurements achieved high predictive accuracy (ICC = 0.887) [17]. Theocharis et al. found that four years of historical data improved balanced accuracy from 67.49% to 91.89% [57]. These findings suggest that disease trajectory information enhances prognostic accuracy and warrants further investigation in future studies.

Our bias assessment revealed a critical gap between model development and evaluation. Thirty‐two of 33 studies trained models on the OAI, which reduced measurement bias through standardised imaging protocols and patient cohorts. However, most studies had high or unclear risk of bias during evaluation due to lack of external validation, small sample sizes, or overfitting. While the OAI's standardisation benefits model development, relying on a single data source introduces substantial selection bias that limits generalisability.

The OAI enroled a cohort that was approximately 79% Caucasian, with participants who were frequently overweight and had existing knee symptoms or risk factors for developing KOA. Critically, it used research‐grade imaging protocols, which differ from those used in routine clinical practice. It recruited younger participants than typical OA populations because it aimed to study progression, not established disease. It followed participants for a maximum of eight years, although KOA progression can span decades [33]. Given these limitations, models trained on this population will likely fail in broader clinical settings.

External validation could reveal these failures, but validation was scarce. Five studies claimed external validation by testing on a held‐out OAI site, which shares the same standardised protocols, quality control, and cohort characteristics and are not truly independent. Of the nine studies (27%) that tested models on independent data, eight used the MOST dataset, which shares some of the OAI's demographic limitations and research protocols. The repeated use of OAI and MOST across studies also raises concerns about benchmark saturation: models may be optimised for data‐specific imaging artefacts and cohort characteristics rather than learning generalisable prognostic features. Externally validated models consistently showed lower AUCs than internally validated ones, suggesting that reported internal accuracy substantially overestimates real‐world performance

Inconsistent reporting made it difficult to evaluate and compare models. Many studies did not share code, and some provided links to empty repositories. Most did not report sufficient architectural details, hyperparameter choices, or training procedures for others to reproduce their work. AUC values were the most reported metric, and while they facilitate comparison across studies, they represent an inherent limitation by providing only a single summary measure. AUC can obscure critical trade‐offs between sensitivity and specificity, potentially masking inadequate performance. For clinical implementation, individual sensitivity and specificity values at specific operating points are essential.

Future research should prioritise developing standardised progression definitions that better reflect the multifaceted nature of KOA. This may involve creating new disease surrogates using data‐driven approaches, incorporating functional measures and expanding the focus beyond structural changes to include patient‐reported symptoms and quality‐of‐life measures. Identifying clinically meaningful patient subgroups and developing tailored predictive models for each could further improve clinical relevance Methodological advances must include more sophisticated multimodal architectures with improved optimisation strategies and incorporation of longitudinal data from multiple time points rather than single baseline assessments. Rigorous external validation using independent and diverse patient cohorts is essential for assessing real‐world performance. Additionally, improved reporting standards and open sharing of code would enhance reproducibility and accelerate progress across the field.

Our study has several limitations. Our literature search was limited to three databases and English‐language publications. Authors were not contacted to clarify missing information. We assessed bias using PROBAST‐AI, which is suitable for prediction models, but it is challenging to measure appropriate sample sizes for DL models. Meta‐analysis was not performed due to the high heterogeneity in study design, outcomes, and methods. Since multiple models were derived from the same studies and datasets, nonparametric tests such as the Kruskal‐Wallis and Mann‐Whitney tests were not appropriate for the statistical testing of differences in AUC between groups. Therefore, we restricted all subgroup analyses to descriptive statistics. Although we categorised progression definitions into four groups, substantial within‐category heterogeneity remains, limiting the interpretability of subgroup median AUCs.

## CONCLUSION

DL models can predict KOA progression from medical images with promising internal performance but are not ready for clinical use. Three critical barriers prevent translation: heterogeneous outcome definitions that prioritise structural changes over patient symptoms, overwhelming reliance on a single dataset, and scarce external validation. Although multimodal and longitudinal approaches show theoretical promise, multimodal fusion has yielded inconsistent benefits in practice, and longitudinal modelling remains underutilised across the field.

## AUTHOR CONTRIBUTIONS

Daniel Rueckert and Florian Hinterwimmer conceived the study. Amna Gillani and Florian Hinterwimmer conducted the literature search, study screening, data extraction, quality assessment, and data synthesis. Klaus Woertler, Michael T. Hirschmann, and Rüdiger von Eisenhart‐Rothe provided clinical insight and interpretation of the findings. Amna Gillani drafted the manuscript. All authors reviewed, revised, and approved the final manuscript.

## CONFLICT OF INTEREST STATEMENT

The authors declare no conflicts of interest.

## ETHICS STATEMENT

The authors have nothing to report. The Clinical Trial no. CRD420251080953.

## Supporting information

PRISMA_2020_checklist.

## Data Availability

The data that support the findings of this study are available from the corresponding author upon reasonable request.
